# The Eosinophil Changes, Efficacy and Safety of Pembrolizumab in Advanced Urothelial Carcinoma Patients with an Older Age and a Poor Performance Status

**DOI:** 10.2147/OTT.S389138

**Published:** 2022-11-05

**Authors:** Nobuki Furubayashi, Akinori Minato, Takahito Negishi, Naotaka Sakamoto, Yoohyun Song, Yoshifumi Hori, Toshihisa Tomoda, Mirii Harada, Shingo Tamura, Hiroki Kobayashi, Yamato Wada, Kentaro Kuroiwa, Narihito Seki, Naohiro Fujimoto, Motonobu Nakamura

**Affiliations:** 1 Department of Urology, National Hospital Organization Kyushu Cancer Center, Fukuoka, Japan; 2 Department of Urology, School of Medicine, University of Occupational and Environmental Health, Kitakyushu, Japan; 3 Department of Urology, National Hospital Organization Kyushu Medical Center, Fukuoka, Japan; 4 Department of Urology, Kyushu Central Hospital of the Mutual Aid Association of Public School Teachers, Fukuoka, Japan; 5 Department of Urology, Miyazaki Prefectural Miyazaki Hospital, Miyazaki, Japan; 6 Department of Urology, Oita Prefectural Hospital, Oita, Japan; 7 Department of Medical Oncology, National Hospital Organization Kyushu Medical Center, Fukuoka, Japan

**Keywords:** urothelial carcinoma, pembrolizumab, age, performance status, immune-related adverse events, relative eosinophil count, neutrophil-to-eosinophil ratio

## Abstract

**Background:**

To evaluate the eosinophil changes, efficacy and safety of pembrolizumab treatment in advanced urothelial carcinoma patients of older age and those with a poor performance status (PS).

**Materials and Methods:**

Consecutive patients with advanced UC who received pembrolizumab after the failure of platinum-based chemotherapy between January 2018 and June 2021 were retrospectively examined.

**Results:**

105 patients (median age, 72 years), 71.4% of whom were men, were enrolled. Patients of ≥75 years of age were considered to be older patients (n=40), and patients with PS ≥2 were considered to have a poor PS (n=10). The objective response and disease control rates were 42.5% and 52.5%, respectively, in older patients and 0% and 10.0%, respectively, in patients with a poor PS. Overall survival (OS) in the older and younger groups did not differ to a statistically significant extent. However, a poor PS was significantly associated with poor survival. Safety analyses demonstrated no significant difference in the occurrence of any immune-related adverse events (irAEs), including grade ≥3, between the older and younger groups. However, a poor PS was significantly associated with the low occurrence of any irAEs. The change of the eosinophil count, the increase of the relative eosinophil count (REC) and the decrease of the neutrophil-to-eosinophil ratio (NER) did not differ to a statistically significant extent between the older and younger groups, but showed significant differences between the poor and good PS (PS 0–1) groups.

**Conclusion:**

Pembrolizumab for advanced UC demonstrated similar changes in the eosinophil count, efficacy and toxicity in both older and younger patients. In patients with a poor PS, although toxicity was significantly lower, survival was significantly worse, and neither an increase in REC nor a decrease in NER were observed, but these values showed significant changes in patients with a good PS.

## Introduction

Platinum-based combination chemotherapy remains the standard first-line treatment for local advanced and metastatic urothelial carcinoma (UC).^1^,^2^ However, in patients with advanced UC, it is generally very difficult to obtain long-term survival with platinum-based combination chemotherapy alone, and there has been no internationally accepted standard of care for a long time.

The advent of immune checkpoint inhibitors (ICIs) has revolutionized the treatment of UC. Currently, two types of ICIs are approved in Japan. Pembrolizumab, anti-programmed death 1 (PD-1) antibody, is approved as second-line therapy for patients who previously received platinum-based therapy and subsequently progressed.^3^ For patients who show either a response or stable disease through their full course of platinum-based first-line chemotherapy, avelumab (anti-PD-L1 antibody), is approved as a maintenance therapy.^4^

In this way, ICIs have been shown to be effective treatments after chemotherapy. In addition, ICIs have been reported be associated with a lower rate of treatment-related adverse events than chemotherapy.^3^ Therefore, clinicians may feel that it is more difficult to decide whether to administer subsequent treatment to patients of older age or patients with a poor performance status (PS) in comparison to younger patients or patients with a good PS (PS 0–1) patients because these populations have not been adequately evaluated in clinical trials and because clinicians may also feel that these patients are unlikely to obtain positive outcomes with subsequent treatment in clinical practice. However, clinicians have to decide to administer treatment when older patients or patients with a poor PS hope to receive treatment because there is no other evidence-based treatment in some cases. Therefore, research has focused on the identification of novel biomarkers for ICIs in a number of malignancies, including UC.^5–8^

Recently, eosinophils have been highlighted as potential biomarkers and have been studied in several malignancies.^9–11^ In patients with advanced UC who receive treatment with ICIs, not only the pretreatment eosinophil counts but also changes in eosinophil counts are associated with outcomes.^12^,^13^ However, the associations between these factors and the outcomes of ICI treatment in older patients or patients with a poor PS in comparison to younger patients and patients with a good PS remain unclear.

In the present study, we retrospectively investigated the efficacy, safety, and the change of the eosinophil count in patients with advanced UC treated with pembrolizumab, and compared the outcomes of older patients and patients with a poor PS to those of younger patients and patients with a good PS.

## Material and Methods

### Patients’ Characteristics

This multi-institutional retrospective study was approved by the Institutional Review Board of the National Hospital Organization Kyushu Cancer Center (2020–90) and the ethics committee of each institution and was performed in compliance with the 1964 Declaration of Helsinki and its later amendments.

We retrospectively examined 125 consecutive patients who received pembrolizumab as anti-PD-1 therapy at 6 institutions from January 2018 to June 2021. Twenty patients were excluded due to a lack of clinical data. All patients were histopathologically diagnosed with UC and showed radiologically-confirmed disease progression after platinum-based chemotherapy.

The following clinicopathological characteristics were investigated: age, sex, Eastern Cooperative Oncology Group (ECOG) performance status (PS), primary tumor site, presence of pathological variants, complete blood count including leukocyte fraction, albumin, presence of liver metastasis, line of treatment, best objective response to pembrolizumab treatment based on Response Evaluation Criteria in Solid Tumors version 1.1,^14^ and grades of immune-related adverse events (irAEs) based on the Common Terminology Criteria for Adverse Events version 5.0.^15^ The neutrophil-to-eosinophil ratio (NER) was calculated by dividing the number of neutrophils by the number of eosinophils, and blood test data, including the peripheral relative eosinophil count (REC) measured at the same time as the complete blood count measurements (before the initiation of pembrolizumab treatment and three weeks later) were collected.

### Statistical Analyses

All statistical analyses were performed using the JMP® Pro, version 15.1.0 software package (SAS Institute, Inc., Cary, NC, USA). According to a previous study, the cut-off value of the hemoglobin concentration was <10 g/dL.^3^ A receiver operating characteristics curve analysis was performed to determine the cut-off value of albumin. Student’s *t*-test was used for the comparison of continuous variables, while Fisher’s exact probability test was used for the comparison of categorical variables. The objective response rate (ORR) was defined as the proportion of patients with a partial or complete response with pembrolizumab. The disease control rate (DCR) was a composite of the ORR and stable disease. Differences between two groups, according to age and PS, in ORR, DCR or the incidence of irAEs were assessed using Fisher’s exact test. Overall survival (OS) was assessed using the Kaplan–Meier method with a Log rank test. Univariate and multivariate Cox proportional hazard regression models were used to predict the significance of associations between the clinical parameters and OS. P values of <0.05 were considered to indicate statistical significance.

## Results

### Patient Characteristics

The baseline characteristics of the 105 included patients and the characteristics of the patients stratified by age (<75 and ≥75) and PS (0–1 and PS ≥2) are presented in Table 1. The median age of the patients was 72 years (IQR, 67–77) years, 40 patients (38.1%%) were at least ≥75 years of age, and 10 patients (9.5%) with PS ≥2. The majority of the patients were male (71.4%), and in the majority of patients the histological type was pure UC (81.0%) and the treatment line of pembrolizumab was 2nd line (80.0%). In the older and younger groups (<75 and ≥75 years), no significant differences were found in factors other than age. Patients with PS ≥2 had significantly lower percentage of male patients (P=0.030), and higher rates of hemoglobin <10 g/dl (P=0.030) and albumin <3.7 g/dl (P=0.002) in comparison to those with a PS 0–1.

**Table 1:** Patient Characteristics According to Age and PS

Characteristics	Total	Age < 75	Age ≥ 75	*P* value	PS 0–1	PS ≥ 2	*P* value
	n=105	n=65	n=40		n=95	n=10	
Age (years), median (IQR)	72 (67–77)	68 (62–71)	79 (77–82)	<0.001	72 (67–77)	74 (62–82)	0.722
Male sex, n (%)	75 (71.4)	49 (75.4)	26 (65.0)	0.273	71 (74.7)	4 (40.0)	0.030
ECOG PS score, n (%)				1.000			<0.001
0–1	95 (90.5)	59 (90.8)	36 (90.0)		95 (100)	0	
≥2	10 (9.5)	6 (9.2)	4 (10.0)		0	10 (100)	
Primary tumor site, n (%)							
Bladder	42 (40.0)	30 (46.2)	12 (30.0)	0.151	38 (40.0)	4 (40.0)	1.000
Upper urinary tract	41 (39.0)	25 (38.5)	16 (40.0)	1.000	37 (38.9)	4 (40.0)	1.000
Upper urinary tract + bladder	22 (21.0)	10 (15.3)	12 (30.0)	0.088	20 (21.1)	2 (20.0)	1.000
Pure UC in histologic testing, n (%)	85 (81.0)	53 (81.5)	32 (80.0)	1.000	75 (78.9)	10 (100)	0.203
Hemoglobin, n (%)				0.088			0.030
≥ 10g/dl, n (%)	47 (72.3)	21 (53.8)	21 (53.8)		65 (69.2)	3 (30.0)	
< 10g/dl, n (%)	18 (27.7)	18 (46.2)	18 (46.2)		29 (30.8)	7 (70.0)	
Albumin (g/dl)				0.291			0.002
≥3.7	70 (66.7)	46 (70.8)	24 (60.0)		68 (71.6)	2 (20.0)	
<3.7	35 (33.3)	19 (29.2)	16 (40.0)		27 (28.4)	8 (80.0)	
Liver metastasis	19 (18.1)	13 (20.0)	6 (15.0)	0.608	16 (16.8)	3 (30.0)	0.383
Treatment line				1.000			0.683
2nd	84 (80.0)	52 (80.0)	32 (80.0)		75 (78.9)	9 (90.0)	
≥ 3rd	21 (20.0)	13 (20.0)	8 (20.0)		20 (21.1)	1 (10.0)	

**Abbreviations**: IQR, interquartile range; ECOG PS, Eastern Cooperative Oncology Group Performance Status; UC, urothelial carcinoma.

### Efficacy

**Figure 1:**
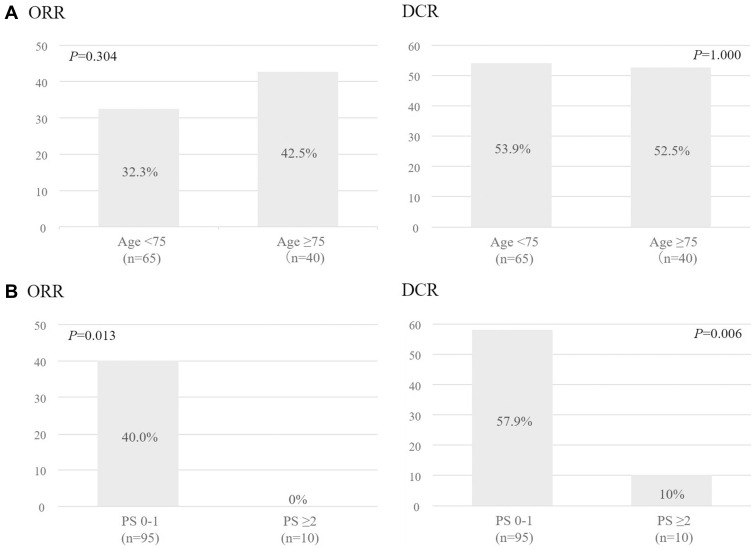
(**A**). Objective response rates and disease control rates according to age (<75, ≥75 years) in patients treated with pembrolizumab. (**B**). Objective response rates and disease control rates according to PS (0–1, ≥2) in patients treated with pembrolizumab.

**Figure 2:**
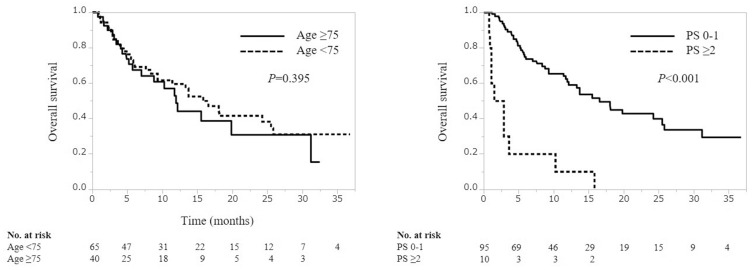
Overall survival according to age (<75, ≥75 years) and PS (0–1, ≥2) in patients treated with pembrolizumab.

The median follow-up period of all patients was 8.4 months (IQR, 4.1–15.7 months). In the entire cohort (ORR, 36.2%; DCR, 46.7%). There were no significant differences in the ORR or DCR between the younger and older groups (ORR: P=0.304, DCR: P=1.000, respectively), but were a significant differences in the ORR and DCR between the PS 0–1 and PS ≥2 groups (ORR: P=0.013, DCR: P=0.006, respectively) (Figure 1A and B). The median overall survival (OS) of the younger and older groups was 15.9 months (95% confidence interval [CI]=9.3–25.5) and 11.9 months (95% CI= 5.7–31.2), respectively. According to the Log rank test, the difference was not statistically significant (p=0.395). The median OS of the PS 0–1 and PS ≥2 groups were 16.6 months (95% CI=11.9–25.5) and 2.2 months (95% CI= 0.8–3.6), respectively; according to the Log rank test, this amounted to a significant difference (p<0.001) (Figure 2).

### Safety

**Figure 3:**
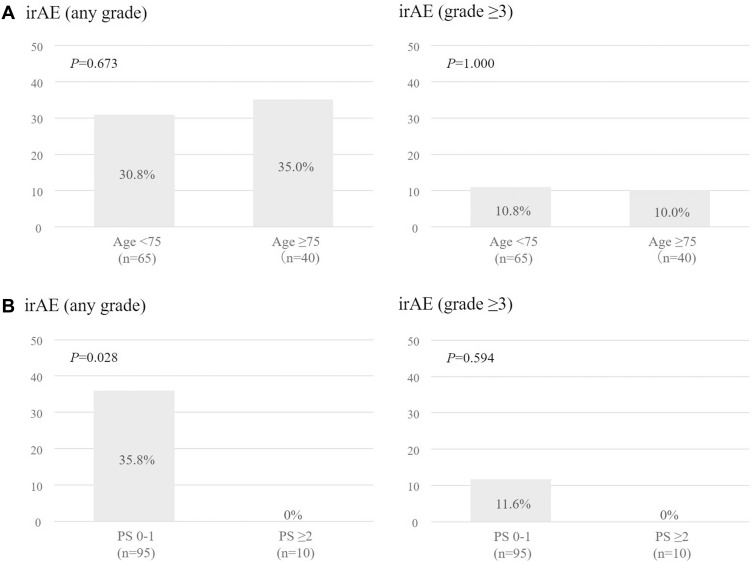
(**A**). Incidence of immune-related adverse events with any grade and grade ≥3 according to age (<75, ≥75 years) in patients treated with pembrolizumab. (**B**). Incidence of immune-related adverse events with any grade and grade ≥3 according to PS (0–1, ≥2) in patients treated with pembrolizumab.

Overall, irAEs of any grade were observed in 34 patients (32.4%), including 11 patients (10.5%) with grade ≥3 irAEs. The rate of any-grade irAEs in the younger and older groups was 30.8% and 35.0%, respectively, which did not amount to a significant difference (P=0.673). The rate of severe irAEs (grade ≥3) in the younger and older groups group was 10.8% and 10.0%, respectively, which was not statistically significant (P=1.000). The rate of any-grade irAEs in the PS 0–1 and PS ≥2 groups was 35.8% and 0%, respectively, which was statistically significant difference (P=0.028). The rate of severe irAEs (grade ≥3) in the PS 0–1 and PS ≥2 groups was 11.6% and 0%, respectively, which did not amount to a significant difference (P=0.594) (Figure 3A and B).

### Eosinophil-Related Change

Before the initiation of pembrolizumab, there were no significant differences in REC or NER between the younger and older groups (P=0.595, P=0579, respectively) or between the PS 0–1 and PS ≥2 groups (P=0.088, P=0.052). A comparison of the REC and NER at 3 weeks after the initiation of pembrolizumab between the younger and older groups revealed no significant differences in REC or NER (P=0.699, P=0.815, respectively), or in the rate of increased REC or decreased NER (P=0.411, P=0.154, respectively). In contrast, when the same parameters were compared between the PS 0–1 and PS ≥2 groups at 3 weeks later after the initiation of pembrolizumab, significant differences were observed in the REC and NER (P=0.003, P=0.002, respectively), as well as in the rates of increased REC and decreased NER (P=0.006, P=0.013, respectively) (Table 2).

**Table 2:** Changes in the Relative Eosinophil Count and Neutrophil-to-Eosinophil Ratio Before and Three Weeks After Pembrolizumab According to Age (<75, ≥75 Years) and PS (PS 0–1, PS ≥2)

Eosinophils	Age			PS		
	Age <75 (n=65)	Age ≥75 (n=40)	*P* value	PS 0–1 (n=95)	PS ≥2 (n=10)	*P* value
Before initiation of pembrolizumab						
REC (%), median (IQR)	2.1 (0.9–4.3)	1.7 (0.8–4.7)	0.595	2.1 (0.9–4.3)	1.0 (0.5–2.4)	0.088
NER, median (IQR)	29.3 (16.2–75.7)	38.5 (13.2–83.0)	0.579	30.6 (13.7–74.4)	76.9 (32.8–160.8)	0.052
3 weeks after initiation of pembrolizumab						
REC (%), median (IQR)	2.8 (1.4–5.2)	3.1 (1.2–5.6)	0.699	3.1 (1.4–5.4)	0.3 (0.1–2.7)	0.003
Increased REC, n (%)	38 (58.5)	27 (67.5)	0.411	63 (66.3)	2 (20.0)	0.006
NER, median (IQR)	23.9 (11.8–50.5)	22.8 (11.5–55.5)	0.815	22.0 (11.4–48.3)	336.3 (30.8–824.5)	0.002
Decreased NER, n (%)	36 (55.4)	28 (70.0)	0.154	62 (65.3)	2 (20.0)	0.013

**Abbreviations**: REC, relative eosinophil count; IQR, interquartile range; NER, neurophil-to-eosinophil ratio; PS, performance status.

### Predictors of OS

The univariate Cox regression analysis revealed that female sex, ECOG PS ≥2, albumin <3.7 g/dl, and the presence of liver metastasis were significantly associated with the prognosis. The multivariate analyses revealed that female sex (hazard ratio [HR] 2.330, 95% CI: 1.271–4.272, P=0.006), ECOG PS ≥2 (HR 3.159, 95% CI: 1.477–6.759, P=0.003), albumin <3.7 g/dl (HR 3.391, 95% CI: 1.835–6.266, P<0.001) were independently associated with the prognosis (Table 3).

**Table 3:** Univariate and Multivariate Analyses of Factors Associated with Overall Survival in Patients Receiving Pembrolizumab

Variable		Univariate		Multivariate	
		HR (95% CI)	*P* value	HR (95% CI)	*P* value
Age	<75 years	1			
	≥75 years	1.268 (0.732–2.196)	0.397		
Sex	Male	1		1	
	Female	2.141 (1.244–3.687)	0.006	2.330 (1.271–4.272)	0.006
ECOG PS	0–1	1		1	
	≥2	6.613 (3.259–13.419)	<0.001	3.159 (1.477–6.759)	0.003
Primary tumor site	Bladder	1			
	Upper urinary tract	1.327 (0.728–2.419)	0.356		
	Bladder + upper urinary tract	1.116 (0.544–2.287)	0.765		
Histology	Pure UC	1			
	Mixed UC	0.750 (0.367–1.534)	0.431		
Hemoglobin	≥10 g/dl	1			
	<10 g/dl	1.566 (0.899–2.726)	0.113		
Albumin	≥3.7 g/dl	1		1	
	<3.7 g/dl	3.408 (1.959–5.929)	<0.001	3.391 (1.835–6.266)	<0.001
Liver metastases	Absent	1		1	
	Present	2.586 (1.415–4.727)	0.002	1.722 (0.918–3.231)	0.091

**Abbreviations**: ECOG PS, Eastern Cooperative Oncology Group Performance Status; UC, urothelial carcinoma.

## Discussion

The present study investigated the change in eosinophils, and the efficacy and safety of pembrolizumab after the failure of platinum-based chemotherapy for advanced UC in older patients (age ≥75 years) and patients with a poor PS (PS ≥2) in comparison to younger patients (age <75) and patients with a good PS (PS 0–1). In this real-world retrospective study, the changes in REC and NER provided by pembrolizumab treatment in older patients were comparable to those in younger patients; however, the significant change in the eosinophil count, the significant increase in REC and the significant decrease in NER, in good PS were not observed in the poor PS group. In addition, according to efficacy profile, the older group showed comparable ORR, DCR and OS to the younger group, while patients with PS ≥2 showed worse ORR, DCR and OS than patients with PS 0–1. According to safety profile, older patients showed comparable any-grade irAEs (including ≥G3) to younger patients, while patients with PS 0–1 showed a worse incidence of any-grade irAEs (including ≥G3) in comparison to the PS ≤2 group.

As a disease of aging, older individuals make up a large proportion of patients diagnosed with cancer. However, older patients are more likely to be excluded from participation in clinical trials, largely due to comorbidities, prior cancer diagnoses, and a reduced functional status.^16^,^17^ Clinical trial data results with a relatively small number of older participants, all of whom were carefully selected for their health, are unlikely to be representative of the general older population. Similarly, patients with a poor PS are unlikely to be incorporated in clinical trials considering the safety profile. Recently, eosinophils have been highlighted as potential biomarkers and have been studied in patients with advanced UC treated with ICIs;^12^,^13^ however, no studies have investigated eosinophils in older patients or patients with a poor PS.

The analysis of patient-reported outcomes in KEYNOTE-045 trial revealed that pembrolizumab significantly prolonged the time to deterioration in health-related quality-of-life in comparison to the investigator’s choice of chemotherapy.^18^ Although pembrolizumab had a more favorable tolerability profile in comparison to conventional chemotherapy, clinicians wonder if they should administer pembrolizumab to older patients or patients with a poor PS because they also feel that such patients are unlikely to obtain positive outcomes with subsequent treatment in clinical practice because older patients and patients with a poor PS have not been fully included and evaluated in clinical trials. Therefore, studies targeting these patients in clinical practice are very important.

Several meta-analyses have been performed to elucidate the impact of age on the efficacy of ICIs. In these studies, 65 and 75 years were the most commonly used cut-off values to differentiate between older and younger patients.^19–25^ One of the largest reported meta-analyses analyzed 34 phase II or III studies containing a total of 20,511 patients to evaluate the clinical efficacy of ICI immunotherapy among different age groups (<65 vs ≥65 years and <75 vs ≥75 years) across various advanced tumor types. ICI therapy could improve the OS and PFS in patients of <65 and ≥65 years of age. Patients of <75 years of age who were treated with ICIs also showed favorable OS and PFS in comparison to the control group, while patients of ≥75 years of age experienced less survival benefit from ICI immunotherapy. However, it also reported that the power may not have been sufficient to draw definitive conclusions about the effectiveness of ICI immunotherapy in patients of ≥75 years of age due to the limited number of patients in this age group.^19^

Life expectancy has been significantly extended, and we thought that it would be more useful—for clinical practice—to define older people as individuals of ≥75 years of age. In the present study, 75 years was used as the cut-off value for age and pembrolizumab for advanced UC provided not only comparable efficacy but also similar toxicity in older patients also in comparison to younger patients. In addition, the change in the eosinophil count, the increase of REC, and the decrease of NER did not differ to a statistically significant extent between older and younger patients. Age-related alterations in the immune system primarily involve the T cell-mediated immune function, which is a crucial aspect of immunosenescence.^26^ Theoretically, age-related immunosenescence in older patients could be considered to have a negative impact on the clinical efficacy of ICIs. On the other hand, it was also reported that chronological age did not comprehensively reflect the of physiological age status of patients.^27^ The gap between chronological age and physiological age may have been responsible for the lack of a difference in efficacy between the age groups in the present study because clinicians had judged that the patients in the older group would be able to tolerate pembrolizumab treatment.

In the KEYNOTE-045 clinical trial, patients with ECOG PS2 and one or more of the established poor prognostic factors for second-line therapy (ie, hemoglobin <10 g/dl, presence of liver metastasis, and receipt of the last dose of most recent chemotherapy <3 months before enrollment) were excluded from enrollment.^3^ Therefore, this clinical trial included only 6 patients (2.2%) with a PS2. In clinical practice, pembrolizumab would be administered to patients with PS ≥2 who wish to receive treatment and who clinicians have judged will be able to tolerate treatment. In other words, these populations represent selected patients with PS ≥2. However, the median OS of patients with PS ≥2 was only 2.2 months, which was significantly shorter than in patients with PS 0–1, whose OS was 16.6 months in the present study. The median time to response in the pembrolizumab cohort was reported to be 2.1 months in the KEYNOTE-045 trial.^3^ Therefore, the patients with PS ≥2 may have died before immunotherapy became fully effective. The present study identified that female sex, PS ≥2, and albumin <3.7 g/dl were associated with a worse prognosis, whereas age ≥75 years was not associated with OS. In addition, a female sex, hemoglobin <10 g/dl and albumin <3.7 g/dl were confirmed to be variable factors that showed a significant difference between the PS 0–1 and PS ≥2 groups.

Interestingly, the incidence of adverse events in patients with PS ≥2 was significantly lower than that in patients with PS 0–1, although in some cases, adverse events may have been judged as symptoms of cancer progression. Several studies, including studies on advanced UC, have reported a possible association between the incidence of irAEs and the clinical efficacy of ICIs.^28–30^ In the PS ≥2 group, the shorter OS may have been due to the lower incidence of irAEs. In addition, interestingly, patients with PS ≥2 did not show a change of eosinophils, increase of REC, and decrease NER, while these values showed significant changes in patients with PS 0–1. It was also reported that the increase in REC and decrease in NER after pembrolizumab were associated with improved OS.^13^ Therefore, the absence of eosinophil changes (eg, increase of REC and decrease of NER) may have also contributed to the shorter OS in the PS ≥2 group in the present study.

The subjects of the present study were patients with PS ≥2 for whom pembrolizumab could be administered after chemotherapy at the clinician’s discretion; patients who chose best supportive care after chemotherapy or who were judged by clinicians to be unable to tolerate pembrolizumab after chemotherapy were excluded. Whether there is a true merit in administering pembrolizumab to patients with PS ≥2 should also be examined in terms of QOL, and include not only patients who desire to receive best supportive care after chemotherapy but also patients are judged by clinicians to be unable to tolerate pembrolizumab. Although clinicians prefer treatments that prolong OS, clinicians should always be conscious of whether the treatment will truly benefit the patient, and should provide the patient with a sufficient explanation before treatment, especially for patients with a poor PS.

The present study was associated with several limitations. This was a retrospective study, and the numbers of patients in the older group and the poor PS group were relatively small, which may have caused a selection bias and affected the results. Furthermore, there was heterogeneity in the lines of prior systemic chemotherapy, pre- and post-treatment regimens, dosing schedule, and the timing at which responses and irAEs were evaluated due to the multi-institutional nature of the study. Further large-scale multicenter studies are required to evaluate the efficacy and toxicity of ICI immunotherapies in older patients and patients with a poor PS.

## Conclusion

In patients with advanced UC who received pembrolizumab after platinum-based chemotherapy, the change of eosinophil did not differ between older (≥75 years) and younger (<75 years), but did differ between patients with PS ≥2 and those with PS 0–1. In older patients, the efficacy and safety of pembrolizumab after platinum-based chemotherapy did not differ from that in younger patients. On the other hand, in patients with PS ≥2 treatment showed worse efficacy in comparison to those with PS 0–1; however, the incidence of irAEs in the PS 0–1 group was higher in comparison to the PS ≥2 group.

## Abbreviations

ICIs, immune checkpoint inhibitors; UC, urothelial carcinoma; PS, performance status; REC, relative eosinophil counts; NER, neutrophil-to-eosinophil ratio; irAEs, immune-related adverse events; ORR, objective response rate; OS, overall survival; IQR, interquartile range.

## Data Sharing Statement

The datasets used and/or analyzed during the current study are available from the corresponding author on reasonable request.
